# Recent trends in core/shell nanoparticles: their enzyme-based electrochemical biosensor applications

**DOI:** 10.1007/s00604-024-06305-4

**Published:** 2024-04-04

**Authors:** Selva Bilge, Burcu Dogan-Topal, Manolya Müjgan Gürbüz, Sibel A. Ozkan, Ali Sınağ

**Affiliations:** 1 Department of Chemistry, Ankara University, 06100 Besevler Ankara, Turkey; 2 Faculty of Pharmacy, Department of Analytical Chemistry, Ankara University, 06560 Ankara, Turkey; 3 Faculty of Engineering, Department of Food Engineering, Istanbul Aydın University, 34307 Kücükcekmece Istanbul, Turkey

**Keywords:** Electroanalysis, Nanomaterials, Core/shell nanoparticles, Enzyme-based biosensors

## Abstract

Improving novel and efficient biosensors for determining organic/inorganic compounds is a challenge in analytical chemistry for clinical diagnosis and research in biomedical sciences. Electrochemical enzyme-based biosensors are one of the commercially successful groups of biosensors that make them highly appealing because of their low cost, high selectivity, and sensitivity. Core/shell nanoparticles have emerged as versatile platforms for developing enzyme-based electrochemical biosensors due to their unique physicochemical properties and tunable surface characteristics. This study provides a comprehensive review of recent trends and advancements in the utilization of core/shell nanoparticles for the development of enzyme-based electrochemical biosensors. Moreover, a statistical evaluation of the studies carried out in this field between 2007 and 2023 is made according to the preferred electrochemical techniques. The recent applications of core/shell nanoparticles in enzyme-based electrochemical biosensors were summarized to quantify environmental pollutants, food contaminants, and clinical biomarkers. Additionally, the review highlights recent innovations and strategies to improve the performance of enzyme-based electrochemical biosensors using core/shell nanoparticles. These include the integration of nanomaterials with specific functions such as hydrophilic character, chemical and thermal stability, conductivity, biocompatibility, and catalytic activity, as well as the development of new hybrid nanostructures and multifunctional nanocomposites.

**Graphical Abstract:**

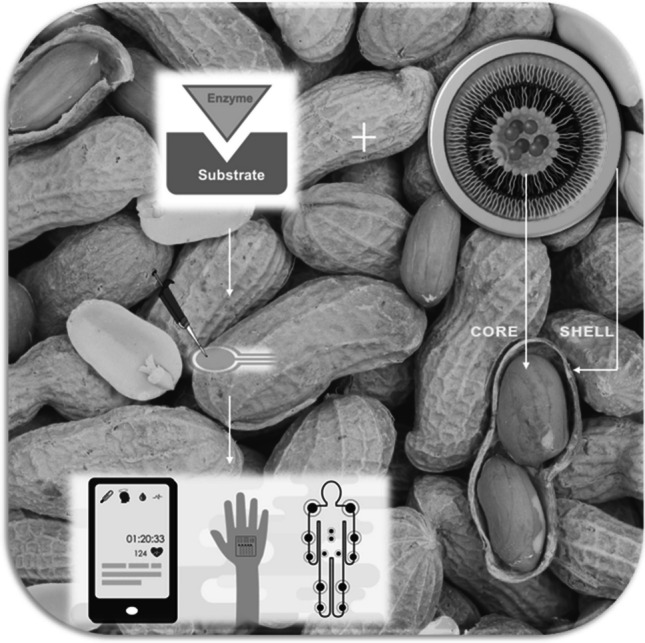

## Introduction

Nowadays, it is well known that nanoscale materials have much more functional, superior, and novel properties than bulk forms. In recent years, nanoparticles became the focus of attention of researchers when it was discovered that the transition from microscale to nanoscale resulted in such drastic changes in material properties. From this point of view, nanotechnology is making tremendous progress in different branches, such as energy storage, sensing, and nanomedicine. The advancement of nanotechnology has led to various nanostructured material forms with different morphology, shape, composition, and surface charge. Nanoparticles consist of a single skeleton, but as the name implies, core/shell materials contain different structures [1].

**Fig. 1:**
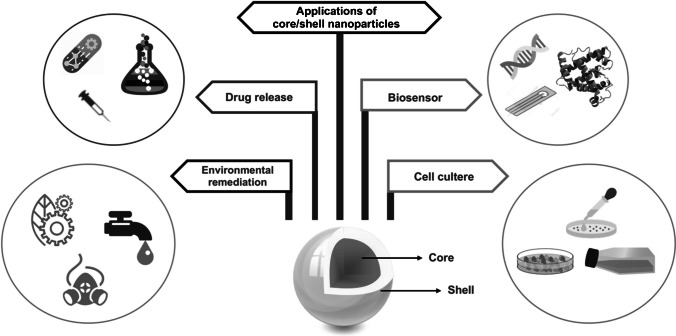
Applications of core/shell nanoparticles

We know that in recent years, the interest in core/shell materials has increased due to the numerous advantages and various usage areas (Fig. 1).

The most important point that distinguishes these materials from basic nanoparticles is that their functionality increases tremendously because they are multilayered. They are preferred in biomedical and sensor applications due to their hydrophilic character, high surface energies, chemical stability, thermal stability, and bio-compatible nature [2]. These structures may have different shapes (hexagon, cube, disk, tube, rod, wire, prism, and octahedron) depending on the synthesis technique and chemical precursors [1]. The effect of nanoparticles in applications is not only dependent on size but also related to shape. Moreover, the catalytic effect, optical properties, selectivity, and electronic surface properties are directly related to the shape. Therefore, the synthesis design of core/shell nanoparticles seriously determines their role in applications. In this context, it is critical to elucidate the structures of synthesized core/shell nanoparticles through various characterization techniques. In addition, the synergetic interaction of the core and shell material is very important in terms of determining the catalytic/electrocatalytic effect mechanism [3]. There are essential factors that determine the synergetic effect: the ligand effect due to the atomic environment affecting the charge, the surface adsorption properties due to the presence of different atomic groups, and the geometric effect caused by the difference in the reactivity of the surface atoms [4].

In its broadest sense, the term “Biosensor” describes a promising and innovative analytical system that includes a wide range of biological sensing elements such as drug analysis, bio-detection, bioengineering, environmental monitoring, military, and security [5]. Biosensors are classified based on biorecognition elements (enzyme, antibody, oligonucleotide, etc.) and transducer (electrochemical, optical, piezoelectric, thermometric). Biosensors have biological recognition surfaces such as enzymes, proteins, DNA, and cells and are analytical devices that can directly convert a biological response into an electrical signal and monitor this signal [6]. Compared to traditional analytical methods, electrochemical biosensors have important advantages such as portability, simplicity, low cost, and eco-friendliness. Real-time or nearly real-time monitoring can be made possible via biosensors, which eliminates the need for repeated sample and laboratory analysis. In terms of consumables and materials used for sample collection, this may result in less waste being produced. Also, biosensors frequently require little energy inputs to function, especially when compared to typical laboratory approaches. This can lead to decreased overall energy use and less environmental impact. The enzyme-based electrochemical biosensors are the ones that attract the most attention from researchers. The first biosensor was developed by Clark and Lyons (1962) as a glucose oxidase biosensor to detect glucose in biological samples [5]. Glucose oxidase biosensors are widely popular in hospitals and diagnostic clinics, as regular monitoring of their blood sugar is essential for chronic diabetic patients. The main advantage biosensors provide in the medical industry is the ability to perform rapid extended analysis at a significantly lower cost per sample [7]. The immobilization of the enzyme on the electrode surface is critical in enzyme-based biosensors and affects the biosensor’s detection performance. Depending on the type of working electrode and enzyme used, the immobilization technique varies. Apart from all these, some critical parameters limit enzyme-based biosensors. These parameters are low enzyme stability, biocompatibility, selectivity, and reproducibility. Moreover, enzyme stability highly depends on environmental factors such as pH, temperature, and humidity [8]. Therefore, developing surface modification strategies to overcome these limitations has become imperative. Enzyme stability is improved by increasing the sensitivity of the electrode surface, mainly through functional nanoparticles. The sensitivity of an electrode surface refers to its ability to detect and respond to changes in the concentration of analytes or target molecules in a sample. Sensitivity is crucial for accurate detection and quantification of the analyte of interest. Among the core/shell nanoparticle types, the preference for magnetic ones as surface modification material provided the control of the enzyme immobilization process and increased the surface sensitivity [9]. When the modification strategies developed in recent years are examined, it is seen that the use of core/shell nanoparticles has increased, and studies on the development of these materials have accelerated. In addition, other non-negligible advantages of core/shell nanoparticles: (i) Due to the structure of the stable shell, it prevents the core nanomaterials from undergoing chemical/physical changes; (ii) The shell not only effectively improves the surface conductivity and surface activity, stability, and dispersion of core nanomaterials, but also provides special optic, electromagnetic, and electrocatalytic performances to the intrinsic ones through surface coating [10]. However, the role of core/shell nanoparticles in enzyme-based biosensor applications is still not fully elucidated. This situation creates a big gap in the literature and is the biggest challenge in front of future studies. In this context, the structural properties, effects, and functionality of core/shell nanoparticles in terms of both core and shell should be examined in depth.

**Fig. 2:**
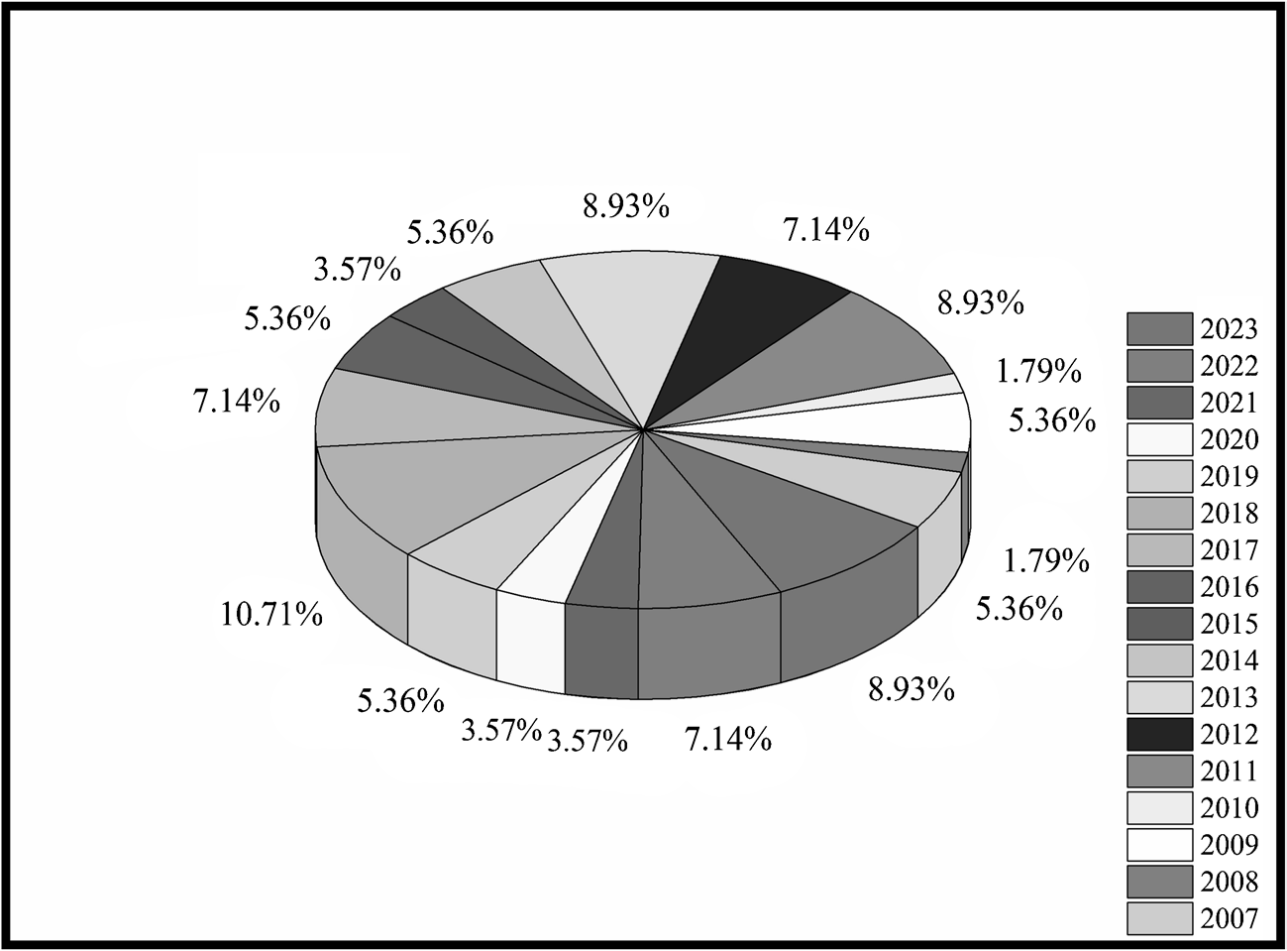
Histogram of the number of publications on core/shell nanoparticles in enzyme-based biosensor applications between 2007 and 2023 (collected from ISI Web of Knowledge, March 2024)

Therefore, it is important to determine the distribution of the number of studies carried out in this field by year and the trend of research groups. When the distribution of publications on core/shell nanoparticles in enzyme-based biosensor applications between 2007 and 2023 is examined, a tendency to increase in the number of publications in this field has been observed, especially in the last 2 years (Fig. 2). Since 2007, the structures of core/shell materials have been better characterized, and their performance as modification materials has been evaluated in more detail. When the reviews in the literature [11]were evaluated, it was determined that only glucose biosensors were emphasized, and the role of core/shell nanoparticles in the electrochemical detection of different substances was not included. Focusing only on magnetic core/shell nanoparticles in the study is insufficient in terms of elucidating the modification properties of other important core/shell nanoparticles. On the other hand, Liu et al.’s study [11]was conducted in 2015 and does not include current studies. In a study conducted by Zhai et al., they focused only on the detection of mycotoxins found in foods [12]. In another study in the literature[13], only Core–shell structured molecular imprinted materials were focused on, and their effects on all biosensors were discussed in general. In this regard, the main objectives of this review are listed as follows:

- It is a detailed examination of the performance of core/shell-based enzymatic biosensors developed in recent years, especially in biological samples, regardless of analyte.
- In-depth examination of the properties of core/shell nanoparticles preferred by researchers in electrochemical enzyme-based biosensor applications and the recommendation of different modification combinations.
- Suggesting and discussing new modification strategies according to the electrode types used in electrochemical applications.
- The advantages and limiting factors of core/shell nanoparticles in enzymatic biosensors are discussed.
- The effect mechanism of core and shell materials on enzymatic biosensors is evaluated separately.
- Information about the synthesis of core/shell nanoparticles is given.

In this study, we focused on the role of core/shell nanoparticles in enzyme-based biosensor applications, the mechanism of these nanoparticles, and the research carried out in recent years.

## Enzyme-based electrochemical biosensors and properties

Biosensors can be classified as *catalytic* or *affinity* systems, depending on the conferring principle of biological selectivity. The enzymes, any cell organelle, or tissue slice showing catalytic activity can be conducted as a biorecognition part in the class of catalytic biosensors [14]. Enzymes, large protein molecules, are macromolecules that catalyze a chemical reaction and do not undergo any changes at the end of the reaction. Using these highly specific structures as a part of the biosensor system means their properties are transferred to the system. The most important of these properties is their high selectivity towards their substrate. Enzymes are used as a biocomponent element in enzyme-based electrochemical biosensors. An enzyme-based biosensor system immobilized on the transducer surface will only be able to receive a signal in the presence of a suitable substrate. The basic principle of amperometric (A) enzyme biosensors is that the analyte undergoes a catalytic reaction by yielding products. The transducer determines the concentration of these products, which is proportional to the analyte concentration [15]. Enzyme biosensors are classified into three main classes: (i) first-generation biosensors, (ii) second-generation biosensors, and (iii) third-generation biosensors based on the electron transfer method [16]. The first-generation biosensors, also called mediatorless A biosensors, measure the concentration of analytes or products of a reaction. These biosensors rely on oxidases and dehydrogenase enzymes, which need coenzymes such as NADH and FADH throughout the catalysis [16]. For example, the glucose oxidase enzyme oxidizes glucose in the presence of oxygen and converts it to gluconolactone by producing hydrogen peroxide and water as a product. In the continuation of the reaction, gluconic acid is formed. GOx needs a redox cofactor called flavin adenine dinucleotide (FAD^+^) to carry out the oxidation process is an electron acceptor that is reduced to FADH_2_ by redox reactions [17]. The reaction that produced H_2_O_2_ using oxygen regenerates FAD^+^ at the anode, which can measure the electron transferred proportionally to the production of H_2_O_2_, hence the amount of glucose present in the blood. The corrections for matrix effects related to interference are often necessary for this type of biosensor [17]. In the second-generation biosensors, also called mediator A biosensors, redox mediators such as ferrocene, ferricyanide, and methylene blue are used as an electron acceptor by replacing oxygen [16]. This approach makes this biosensor possible to work at low potentials. However, these biosensor types are less commonly used than the first-generation due to their low stability because of the immobilized mediators.

The third-generation biosensors consist of the enzyme, redox polymer, and electrode. This biosensor can exchange electrons between the enzymes and the electrode without a mediator. The lack of need for mediators in this type of glucose biosensor has increased selectivity [16]. Enzyme biosensors are commercially successful bioanalytical devices that display high sensitivity and selectivity. In the market, approximately 85% of biosensors are enzyme biosensors. Different modification and immobilization approaches are considered in this manuscript to develop the bioanalytical performance of enzyme biosensors, such as sensitivity, selectivity, and stability.

### Immobilization techniques in brief

Enzyme immobilization is a critical feature in designing the biorecognition component of enzyme-based biosensors [18]. Enzyme immobilization and modification materials are used to improve parameters such as sensitivity, reproducibility, wide linearity range, selectivity, and short response time. For this reason, most researchers carry out intensive studies on immobilization strategies. The electrode and the preferred enzyme type should be considered in the enzyme immobilization process. Some enzyme immobilization procedures employ a variety of immobilization techniques. An enzyme can be pre-immobilized on beads via affinity, adsorption, or covalent bonding before being retained in a porous polymer [18]. Well-known immobilization techniques include electrostatic interactions, physical adsorption, layer-by-layer deposition, electrochemical doping, pre-immobilization on ion-exchanger beads, cross-linking, covalent immobilization, chemisorption, encapsulation, entrapment, and affinity, respectively [19]. The advantage of immobilization techniques based on adsorption is that they do not contain any steps that disrupt enzyme activity or active site and are straightforward to apply. Therefore, they are more in demand from researchers than other immobilization techniques. However, other proteins or substances can do non-specific adsorption together with enzymes. Therefore, at this point, the selectivity of the modification agent used is of great importance.

On the other hand, one of the drawbacks of this technique is the low stability of enzyme storage. This is because the enzyme binds poorly to the electrode surface depending on environmental conditions (temperature, pH, and humidity). Another popular technique for enzymatic biosensors is covalent immobilization. This immobilization technique is chemical-based and consists of a surface binding procedure by the functional groups contained in biocatalysts. First, the surface is activated with reagents such as carbodiimide or glutaraldehyde so that enzymes can bind to the solid support, followed by enzyme binding to the activated support and removing excess/unbound biomolecules. The most significant advantage of the covalent immobilization technique is that it does not damage the enzyme activity and the active site, as in the adsorption-based technique. However, the drawback of this technique is that it causes the enzyme activity to decrease by changing the conformation of the enzyme and affecting the active site of the enzyme. The enzyme immobilization strategy is a critical process that directly affects biosensor efficiency. In this respect, modification strategies developed through core/shell nanoparticles gain significant importance. For instance, Au shell structures are known to be effective in preventing the loss of enzyme molecules. In addition, when the core is made of Au nanoparticles, it has been discovered that the core material facilitates an electrical connection between the enzyme redox regions and the electrode [15]. At this point, it is thought that the design of core and shell materials, considering their specific properties and their use as a biosensor platform, will contribute significantly.

## Overview of core/shell nanomaterials

**Fig. 3:**
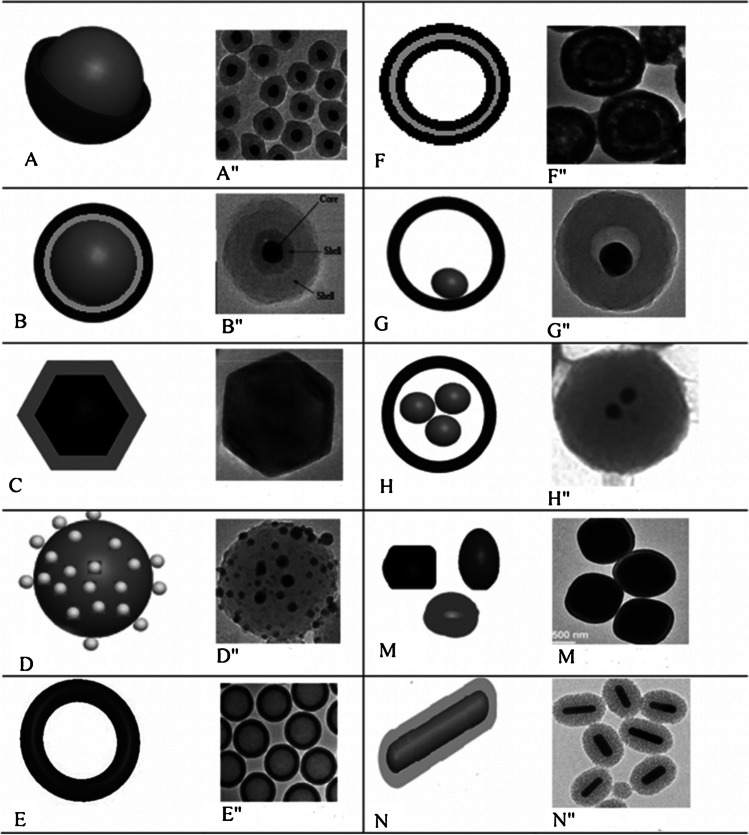
Classification of core/shell nanoparticles according to different morphologies. **A** core/shell NPs, **B** core double-shell particles or core multi-shell NPs, **C** polyhedral core/shell NPs, **D** core porous-shell NPs, **E** hollow-core/shell NPs or single-shell NPs, **F** hollow-core double-shell NPs, **G** moveable-core/shell NPs, **H** multi-core/shell NPs, **M** irregular shape core/shell NPs, **N** rod core/shell NPs. Reprinted from Ref [21]. with permission from Wiley

In recent years, groundbreaking research and rapid advances in nanotechnology have expanded the applications of nanomaterials. With the clarification of the physicochemical properties of these enormous materials, they are now preferred, especially in electrochemical biosensor applications. Moreover, researchers have begun approaching traditional nanomaterials from a new perspective in the last few years. Thus, nanomaterials, including noble metals, which generally act as active catalytic components, can now be synthesized easily and quickly with well-defined sizes, crystal surfaces, shapes, skeletons, and composition [4]. The ability to control the critical properties of nanomaterials to this extent is desirable in many applications in chemistry. Also, it creates a potential for sustainable and green chemical processes. In general terms, core/shell nanoparticles are composites formed with inner layer material (core) and outer layer material (shell), both at the nanoscale [20]. Traditionally, core/shell nanomaterials consist of multilayer and concentric semiconductor materials in connection with the historical development of research. However, the definition of core/shell nanomaterials can be expanded depending on the coating of different boundary materials (either completely or partially) or different inner components. Therefore, core/shell nanostructures can be classified into many classes based on the chemical types and properties of the two components: organic/inorganic, organic/organic, inorganic/inorganic, inorganic/organic, and types [4]. As interest in task-specific core/shell nanostructures increases, component diversity is increasing day by day, and this classification is expanding. For example, in the classification proposed by Gawande et al. [4], materials were classified as hollow core/shell, core-multi shell, and core-porous shell according to their shell structures (Fig. 3**)**.

### Synthesis of core/shell materials

Core/shell nanoparticle synthesis requires a variety of advanced processes, each precisely intended for producing exact structures with specialized attributes suited for a wide range of applications across multiple disciplines. Seed-mediated growth is currently one of the most preferred important techniques [22]. Within the framework of this technique, nanoparticles that function as seeds or cores offer nucleation sites to control the deposition of shell ingredients [22]. The fabrication of well-defined core nanoparticles is normally the first step in the synthesis process. This is then followed by the introduction of precursor molecules to produce the shell material [23]. Researchers can precisely regulate the growth of the shell layer by carefully altering reaction parameters such as precursor concentrations, reaction temperature, and duration. This allows them to produce core/shell nanoparticles that are homogeneous in size, shape, and composition. This technique provides an outstanding degree of adaptability since it enables the insertion of a wide variety of materials into the core and shell. These materials include metals, metal oxides, semiconductors, and polymers, among others [24]. Furthermore, the sequential layer-by-layer deposition technique offers an alternative method for producing core–shell nanoparticles. This approach involves depositing consecutive layers of complementary functional groups or materials onto the core material using electrostatic interactions, hydrogen bonding, or other specialized chemical interactions. By carefully selecting the materials for each layer and managing the deposition process, researchers may precisely adjust the shell’s thickness, composition, and usefulness. Layer-by-layer deposition enables the creation of multifunctional core/shell nanoparticles for a wide range of applications, including catalysis, sensing, drug administration, and biomedical imaging [24, 25]. Chemical vapor deposition (CVD) is another effective method of creating core/shell nanoparticles. In CVD, precursor gases are injected into a reaction chamber and chemically react to deposit thin films on the surfaces of core nanoparticles scattered over substrates. The primary advantage of CVD is its ability to provide exact control over shell thickness, composition, and crystallinity by altering precursor flow rates, temperature, pressure, and reaction time. This allows for the creation of well-defined core/shell structures with specialized characteristics appropriate for specific applications. Furthermore, CVD is scalable and reproducible, making it a viable option for large-scale synthesis of core/shell nanoparticles for industrial applications[4, 26]. Emulsion polymerization serves as an additional flexible methodology in the synthesis of core/shell nanoparticles. By employing this technique, molecules of an emulsion comprising monomers, surfactants, and initiators are utilized to disperse core nanoparticles [27]. Core/shell nanoparticles are produced when a shell of polymer material envelops the core nanoparticles as polymerization continues. One notable benefit of emulsion polymerization is its capacity to generate core/shell nanoparticles, wherein the shell thickness, composition, and morphology can be precisely manipulated [28]. Moreover, this approach provides adaptability in terms of integrating diverse biomolecules or functional groups into the shell, thereby enabling the fabrication of core/shell nanoparticles with multiple functions for biomedical purposes [29, 30]. The sol–gel technique presents an additional flexible pathway for the synthesis of core/shell nanoparticles. Precursor molecules encounter hydrolysis and condensation reactions in this procedure to generate a sol or gel encircling the core nanoparticles. The sol or gel undergoes further solidification due to thermal treatment or aging, which results in the development of a shell encircling the core nanoparticles. The ability to produce core–shell nanoparticles with a significant degree of control over shell thickness, composition, and porosity is the primary benefit of the sol–gel method. Moreover, this approach provides adaptability in integrating diverse dopants or functional groups into the shell, thereby enabling the fabrication of core–shell nanoparticles that possess customized characteristics to suit applications [31–34].

## Recent developments and applications

In the table showing the electrochemical studies carried out in the last years, there has been an increase in studies conducted with the core/shell. The table shows that the most preferred ones are the A method for enzyme-based electrochemical techniques, GOx for enzymes, glucose for the analyte, iron, gold (Au), and silicon dioxide-containing materials for core/shell modification agent, physical adsorption for immobilization technique, and GCE for working electrode and aqueous solutions in applications. Although many techniques are used within the literature, many other practical methods are used, such as enzymes, core/shell modification materials, immobilization techniques, working electrodes, and applications. Therefore, this method can be applied in many areas easily. Additionally, these methods provide a very low limit of detection. The reason for this and increased electrical conductivity is that surface modification can enhance sensitivity and selectivity.

The biosensor designed by Farshchi et al. [35]for monitoring miRNA-21 that graphene quantum dots (GQD) were prepared on conductive nano-ink using Ag@Au core/shell nanoparticles electrodeposited, and caspase enzyme immobilized on the modified surface. The chronoamperometric (CA) method was preferred as the electrochemical technique for detection. The surface area of this nano-ink is excellent for immobilizing biomarkers. The created paper-based biosensor exhibits excellent stability and sensitivity in addition to being incredibly compact and inexpensive. Moreover, it is a paper-based biosensor; therefore, it is cheap and has high sensitivity and stability. Under optimal conditions, the linearity range was found to be between 5 pM and 5 mM, while the detection limit was found to be 5 pM. It was concluded that the biosensor maintained its performance for 48 h thanks to the Ag@Au core/shell nanoparticles prepared in the study.

**Fig. 4:**
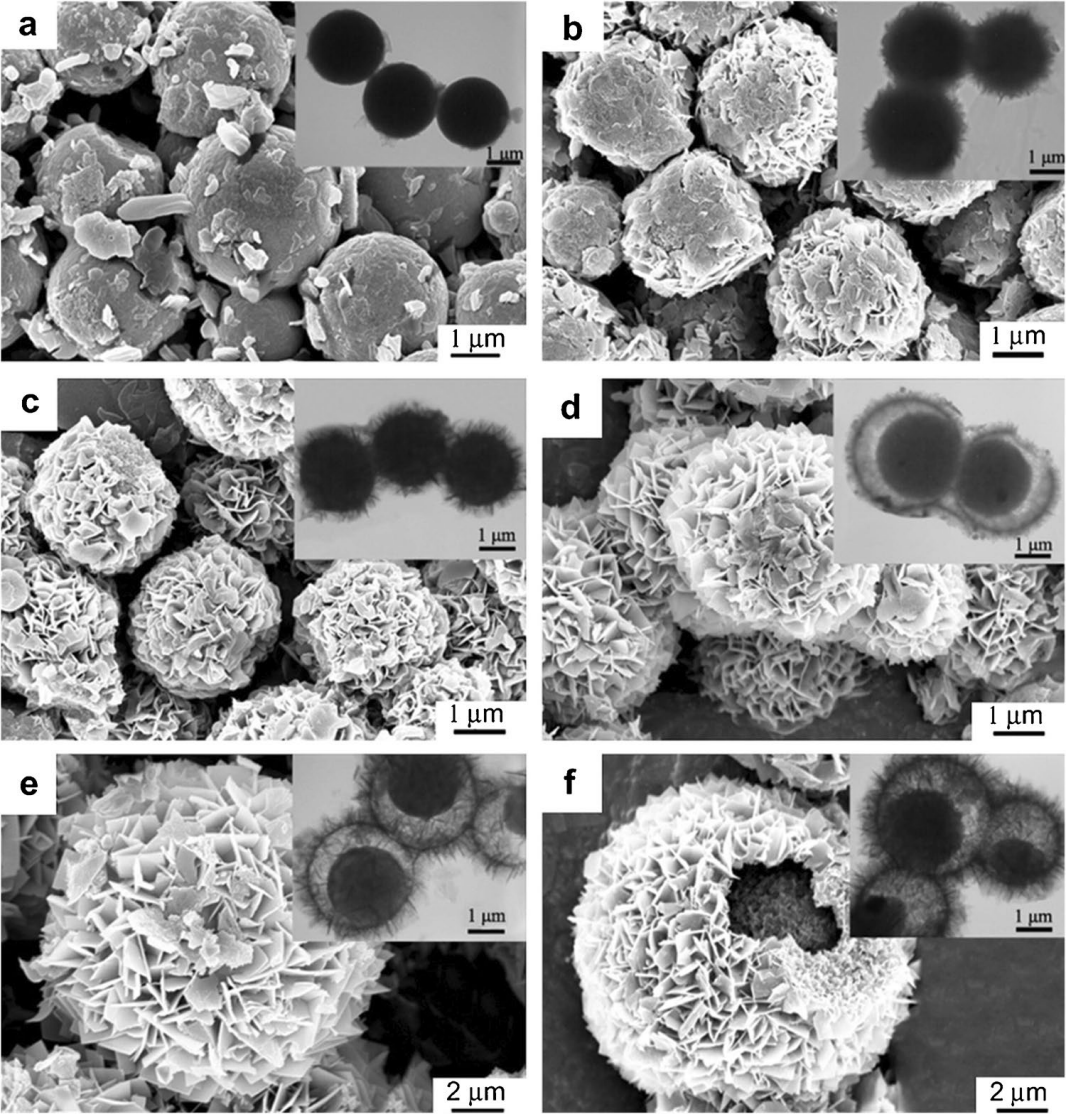
SEM and TEM (additions) images of the calcined TiO_2_ microspheres after observing with different hydrothermal reaction times: 1 h (**a**), 3 h (**b**), 6 h (**c**), 12 h (**d**), 48 h (**e**), and 96 h (**f**). Reprinted from Ref. [36] with permission from Elsevier

In this study, Xie et al. [36] synthesized nanosheet-based titania microspheres with a hollow core/shell structure (Fig. 4) and coupled them to GCE with the help of Nafion to produce a mediator-free biosensor. This material can provide biological compatibility, high conductivity compared to other similar materials, and an eco-friendly and chemically and thermally stable medium for the immobilization of enzymes. The hollow core/shell structure is not found at TiO_2_-1, TiO_2_-6, and D-TiO_2_-48. Despite these, the hollow core/shell structure is found at the TiO_2_-48-based biosensor, which has a more comprehensive linear range. TiO_2_ has pores like “trumpet” shaped between the nanolayers, which are found wider outside the shell. This structure directs the adsorbed that can be immobilized enzymes to inside the core of the microsphere. Furthermore, TiO_2_ increases the strength of the enzyme to leak throughout a reaction with the substrate. The substrate can easily reach the immobilized enzyme inside the hollow core/shell structure. The substrate and enzyme concentration in this narrow region increases the opportunity for interaction. Nanolayer-based TiO_2_ microspheres with hollow core/shell structures can be available to effectively retain other redox-active proteins. These structures have broad applications such as bioelectronics, biosensors, biomedical devices, and biocatalysis. According to spectroscopic and electrochemical analyses, TiO_2_ microspheres provide good enzyme stability and bioactivity by acting as an immobilization support that is biocompatible with enzymes. The resulting biosensor demonstrated good performance for the detection of H_2_O_2_, with both a low detection limit of 0.05 µM and a large linear range of 0.4–140 µM. This is due to the nanosheet-based hollow core/shell structure of the TiO_2_ micro-spheres, which facilitates the direct electron transfer of HRP.

In the study of Villalonga et al. [37], a disposable A biosensor was made for quantification and rapid detection of Brettanomyces bruxellensis (Brett) in red wine. The core/shell structure was modified with Fe_3_O_4_ and SiO_2_ superparamagnetic nanoparticles on SPE, and Con A was immobilized to the surface by covalent bonding. These sensors also applied to real wine samples, demonstrated great performance in terms of linear response range, repeatability, selectivity, stability, and detection limit. It has been indicated in this study that proper functionalization of nanoparticles combined with the use of disposable electrodes can be important in preparing disposable, susceptible, reliable, and electrochemical devices for microbial analysis of food. At the end of this study, a linear range of 10–106 CFU/mL, and a limit of detection of 5 CFU/mL was found, respectively.

In the study of Cui et al.[38], GCE was modified to the core/shell structure, which formed with Au nanorods (AuNRs) and mesoporous SiO_2_ (MS). By combining the suspension of AuNRs@MS core/shell nanoparticles with TiO_2_ precursor solution immediately before being cast on the electrode for gelation, the nanoparticle suspension was doped in mesoporous TiO_2_-CS hydrogel. The TiO_2_-CS hydrogel has a uniform distribution of AuNRs@MS nanoparticles throughout. The electro-conductivity of the TiO_2_-CS hydrogel and the electrocatalytic activity of the AChE immobilized CS/TiO_2_-CS matrix is markedly increased upon doping AuNRs@MS nanoparticles. In addition, AChE was immobilized by physical adsorption for the detection of pesticides by the electrochemical impedance spectroscopy (EIS) method. As a result, this biosensor demonstrated high reproducibility and accuracy in detecting pesticide-added vegetable juice samples. Therefore, the developed AChE biosensor can be shown as a method that can be an important application for the detection of pesticides with high reliability, simplicity, and speed. As a result of this study, the linearity range was found to be between 0.018 and 13.6 μM, while the limit of detection was found to be 5.3 nM.

In this study by Li et al.[39], the Fe_3_O_4_@SiO_2_@vmSiO_2_ microsphere modified on GCE for the detection of dopamine was attached to the surface and stabilized by a covalent bond. The laccase was then immobilized to this modified surface. Detection was made by the EIS method. Fe_3_O_4_@SiO_2_@vmSiO_2_ microspheres show a well-defined core/shell structure with high magnetization, regular microchannel, and lamellar spacing. This developed sensor exhibited excellent selectivity and detection performance. The results of this method, which was used for the detection of dopamine in an aqueous solution, showed a linearity range between 1.5 and 75 µmol L^−1^, while the detection limit was found to be 0.177 µmol L^−1^, which proves that very small-scale measurement could be made.

In the study of Nguyet et al. [40], a new biosensor was made by using diamine oxidase immobilized with the help of covalent binding on CeO_2_-NR@Ppy nanocomposite with [Fe (CN)_6_]^3−/4−^ redox probe to be used in Salmonella detection. Measurements were made using the EIS method. It was found that using this prepared DNA biosensor in Salmonella screening saves time and reduces costs and can be widely used as a powerful tool for food analysis and diagnostic application for Salmonella screening. In this study, the linearity range of the DNA biosensor tested in an aqueous solution was between 0.01 and 0.4 nM, while the limit of detection was found as 0.28 nM.

**Fig. 5:**
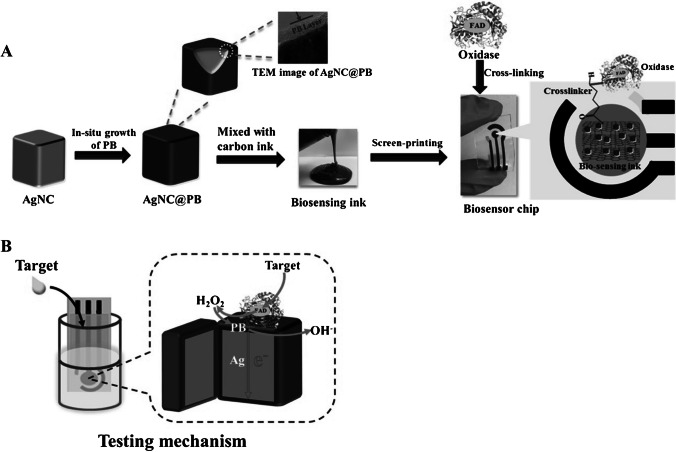
**A** Schematic illustration of the flexible chip based on AgNCs@PB nanocomposites fabricated process. **B** Schematic illustration of the testing mechanism of the flexible chip. Reprinted from Ref [41] with permission from Elsevier

To meet the urgent requirements of practical application in blood sugar, a biosensor with real-time, portable, ultra-high sensitivity and selective biosensing is required. In this study by Yang et al.[41], 3D AgNCs@PB core/shell material was modified on an SPE (Fig. 5). The determination was made by the CA electrochemical method. Glucose is immobilized on this core/shell material by cross-linking. This chip makes glucose measurement approximately four times more sensitive than other SPE electrodes and has great anti-interference features. In this study performed on a rabbit serum sample, the linearity range was found between 0.01 and 1.3 mM, and the detection limit was 0.005 mM.

In this study by Ma et al. [42], the core/shell structured PtPd@NCS nanocomposite was modified on 3DGNE, and then AChE was immobilized with covalent binding. This biosensor was tested, and its scale of detection for parathion methyl, chlorpyrifos, and malathion in potato, corn, and grain samples was determined. The electrochemical method used in this study was amperometry. The developed biosensor exhibited high selectivity, reproducibility, and stability. It also exhibited acceptable recovery when applied to real samples and showed excellent potential in creating biosensors to detect organophosphate pesticides and other analytes. Linearity ranged from 1 × 10^–14^ to 1 × 10^−10^ M, and 1 × 10^−9^ to 1 × 10^−5^ M, and the limit of detection was 7.9 × 10^–15^ M for malathion; linearity ranged from 1 × 10^–13^ to 1 × 10^−6^ M and the limit of detection 7.1 × 10^–14^ M chlorpyrifos; and for parathion methyl, linearity ranged between 1 × 10^–14^ and 1 × 10^–11^ M, and between 1 × 10^−10^ and 1 × 10^−5^ M, and detection limits were at 8.6 × 10^–15^ M.

**Fig. 6:**
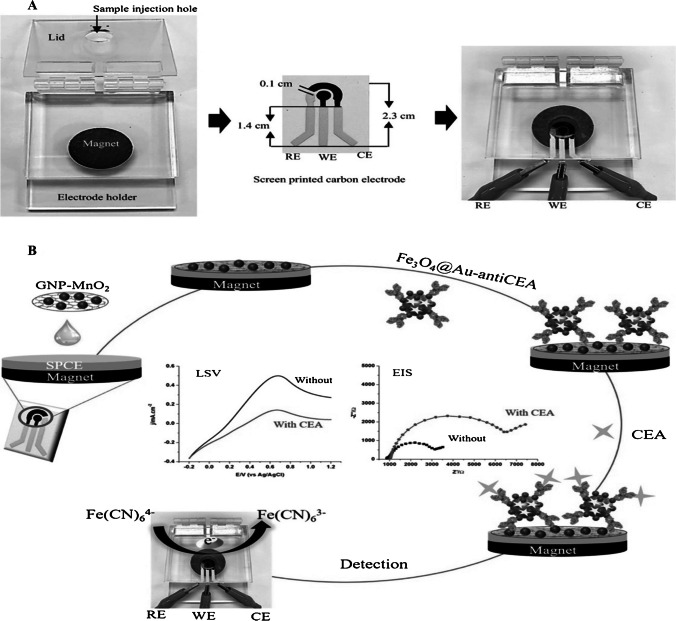
**A** The electrode holder set-up and SPCE, (B) schematic demonstration of the electrochemical sensor fabrication steps for detection of CEA.4P. Reprinted from Ref. [43]with permission from Elsevier

In this study by Butmee et al. [43], for the detection of carcinoembryonic antigen (CEA), SPCE was modified with GNP-MnO_2_ core/shell Fe_3_O_4_@Au nanoparticles on the working electrode (Fig. 6). Upon this modification, anti-CEA was immobilized by covalent bonding. Measurements were taken by LSV and EIS electrochemical methods with the help of a redox probe prepared with 0.1 M phosphate buffer solution containing 5 mM [Fe (CN)_6_]^3−/4−^. The applicability of this prepared immunosensor was verified by applying it to a diluted human serum with the electrochemiluminescence (ECL) method. The results proved that accurate results could be obtained in real sample analysis. It can also be a new diagnostic platform with acceptable accuracy and precision, with high sensitivity and portability at low cost. As a result of the measurements, the linearity range was found between 0.001 and 100 ng/mL, while the detection limit was found to be 0.10 pg/mL for LSV and 0.30 pg/mL for EIS.

In this study by Keerthi et al. [44], a core/shell structure was created by using Mo NPs@f-MWCNTs on SPCE for the detection of dopamine (DA), which was examined by A technique using a redox probe of 5 mM K_3_[Fe (CN)_6_]^3−/4−^ and 0.1 M KCl. Designed by combining the advantages of MWCNT and Mo NPs, the DA biosensor platform offers outstanding electron transferability, good electrocatalytic properties, excellent conductivity and selective and rapid reaction to DA, broad linearity range, low limit of detection, and great selectivity. Furthermore, the developed DA sensor shows good reproducibility and stability. The detection of DA has confirmed the real-time application of this developed sensor. Moreover, it was proven that this method has a very low detection scale with a 1.26 nM in the detection limit.

## Role of core/shell nanomaterials in enzymatic biosensors

Core/shell materials are primarily used in enzyme-based biosensor applications due to their interesting and superior properties. Core/shell structures can exhibit many new characteristics that single nanoparticles do not have and offer much broader opportunities in electrochemical application than single nanoparticles. Primarily, the presence of the relatively stable shell can hold back internal nanomaterials that suffer from chemical/physical changes. Also, the outer shell effectively increases the conductivity, activity, stability, and differentiation of the core nanomaterial while also imparting very important properties such as electromagnetic, optical, and electrocatalytic properties to the core materials. The use of core/shell nanomaterials to immobilize and adsorb enzymes significantly affects critical parameters such as electron transfer activity and the bio-catalytic performance of enzymes [87].

When the existing studies in the literature were examined, it was determined that the characterization techniques applied for synthesizing core/shell materials were insufficient. Therefore, significant gaps have emerged in explaining the mechanism of action of such functional materials. To overcome these challenges, basic characterization techniques such as TEM/SEM and important techniques such as size distribution, EDX mapping, and core-level XPS should be utilized. It is very important to apply size distribution analysis, especially in materials where the core and shell consist of carbon [88].

For example, in a study conducted by Geng et al. [89], a series of multifunctional shape-controlled non-spherical hollow porous silica nanoparticles (HMSNs) were synthesized as drug carriers using Fe_2_O_3_ with four different morphologies such as capsule, cube, rice and rhombus, and multifunctional cap as an encapsulating shell. To elucidate the structure of the materials obtained in the study, high-resolution TEM, BET, XRD, FT-IR, size distribution, ^13^C NMR, and UV–Vis analyzes were used. Thanks to the high-resolution TEM images, it was determined that the outer silica shell contains a relatively regular vertical porous channel due to the CTAB structure orientation effect.

**Fig. 7:**
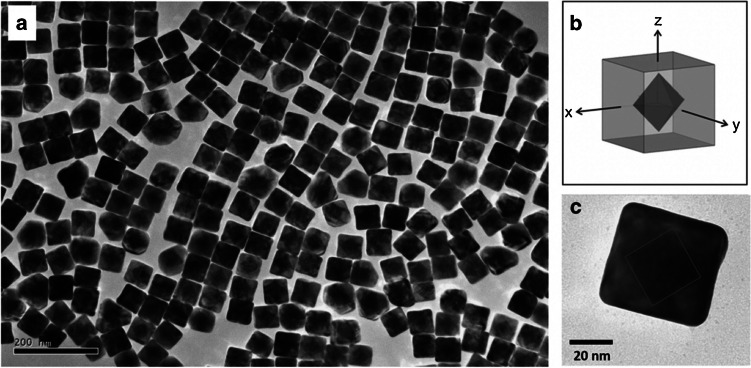
**a** Scanning/transmission electron microscopy analysis results for Au–Pd core/shell material, **b** schematic representation, **c** image of a single nanoparticle. Reprinted from Ref. [90] with permission from Nature.

In a study by Li et al. [90], the illumination of the core/shell structure was performed by X-ray free electron lasers (XFELs) analysis, which is used to determine the structures of non-crystalline single molecules or nanoparticles from coherent diffractive imaging data. In the study, Au–Pd core/shell particle (Fig. 7) was used as a model system to study XFEL scattering patterns.

**Fig. 8:**
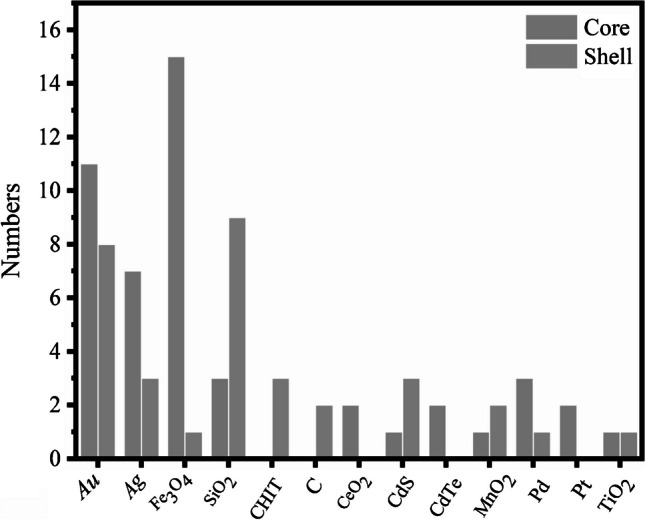
Statistical grouping of core and shell components preferred in enzyme-based biosensor applications

It is critical to assess the properties of the core and shell independently to understand the effects of these materials on biosensing processes. Figure 8 shows the statistical grouping of core and shell components preferred in enzyme-based biosensor applications. The results show that Fe_3_O_4_, Au, Ag, and Pd are mainly used as core material types, while SiO_2_, Au, CHIT, and CdS are used as shell material types.

### Role of the core in enzymatic biosensors

**Fig. 9:**
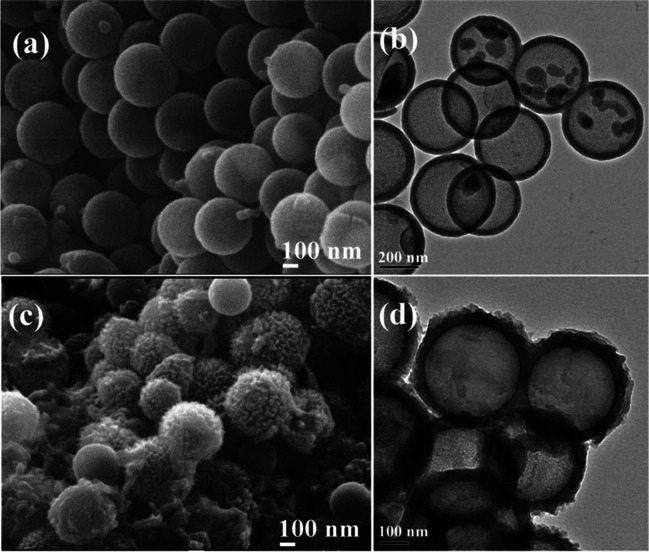
SEM (**a**, **c**) and TEM (**b**, **d**) images of HCS (**a**, **b**) and HCS@PANI (**c**, **d**) nanocomposites. Reprinted from Ref. [91] with permission from Elsevier

At this point, it should not be forgotten that the morphology of the carbon material is an element that determines the core function. Hollow carbon spheres are particularly interesting in hollow nano/microstructure materials due to their unique structure, low density, large surface area, thermal resistance, and electronic properties. For example, in a study conducted by He et al. in 2018, hollow carbon spheres coated with needle-like polyaniline (HCS@PANI) were used [91]. Figure 9 shows TEM images of the nanocomposite. The achievement of the HCS@PANI nanostructure, the advantage of the hollow nanostructure, amino efficiency, and improved electrochemical activity endowed it with a strong affinity towards AChE and resulted in a stable electrochemical inhibition biosensor capable of quantitatively detecting malathion [91].

On the other hand, in the literature, some studies [54] suggest that a hollow carbon core can accelerate enzyme–substrate activation. The main reason for this may be the stability, hydrophilicity, and biocompatibility features of hollow core structures.

In addition, Li et al. [53] examined the advantages of the spherical structure. In general, Au nanoparticle/PANI hybrid composites are mostly prepared by random doping or embedding of Au nanoparticles into conductive polyaniline film. However, this study indicates that Au/polyaniline core/shell materials provide far superior properties and advantages in enzyme-based biosensor research. In particular, it was predicted that the spherical structured Au core component could facilitate the electrical contact of the enzyme redox sites with the electrode [53].

In many studies, it has been concluded that Au nanoparticles should be used together with magnetic nanoparticles. Because the magnetic nanoparticles can be easily separated from the liquid phase by a magnet and immediately redispersed with the removed magnet, this makes it possible to carry out the modification and hybridization process away from the electrode surface, greatly helping to avoid non-specific adsorption in the sensor. In addition, cores formed from Au-magnetic nanoparticles are also effective in reducing surface oxidation [52].

Semiconductor-core/shell nanostructures are considered one of the most suitable methods for charge separation. They are synthesized by combining two or more semiconductors with a suitable lattice pairing between them. Among them, CdTe-CdS is an interesting core/shell quantum dot with ultra-fast charge carrier and charge transfer capability. Especially in glucose detection, the synergetic electrocatalytic effect and electron transfer efficiency of this material draw attention [48].

It should be noted that in enzyme-based biosensor studies, alternative core structures such as graphene or quantum dots are waiting to be discovered. For instance, in a study conducted by Paimard et al. [92], the preference for honey as the core showed an increase in adhesion on the nanofibers on the electrode surface. Honey is preferred for producing electrospinning nanofibers due to its antimicrobial, anti-inflammatory, and antioxidant properties along with various polymers [92].

### Role of the shell in enzymatic biosensors

A vital synthesis strategy is to determine shell materials by considering critical parameters for sensor applications such as aggregation, biocompatibility, and conductivity. In addition, shell materials’ properties significantly affect core materials’ functionality. Considering that core materials are generally magnetic particles, bare magnetic particles are prone to aggregation and oxidation [11]. It is an effective way to synthesize magnetic nanoparticles with stable and robust properties and to use shell materials to coat/decorate the magnetic core. In this respect, the tasks of the shell material can be summarized as follows: (i) The shell materials not only prevent the oxidation of O_2_ of the magnetic cores but also increase the stability of the magnetic core/shell nanocomposites. (ii) Shell materials can embed many functional materials with more active sites, biocompatibility, and specific recognition sites on the surface of magnetic nanoparticles. (iii) Since polymer-based shell materials contain many active groups such as carboxyl, hydroxyl, sulfo, and sulfhydryl group, they offer many active sites to magnetic nanoparticles and can improve the mechanical and optical properties of magnetic nanoparticles [11].

On the other hand, evaluating the shell materials in themselves is necessary. Compared with other shells, carbon-structured shells have higher stability in extreme conditions such as high temperature, pressure, acidic, or basic. Moreover, the outer polysaccharide shell presents various functional groups, such as carboxylic, aldehyde, and hydroxyl. The carbon structure’s high surface area catalyzes the electrochemical charge transfer acceleration in the solution environment [78].

In some studies, it is noteworthy that materials such as Ag, Au, and Pt are used as shells. Nanoparticles with a biocompatible Au shell structure are highly efficient in non-corrosive biological conditions and can be easily functionalized with Au–S chemistry. The Au shell structure is predicted to effectively improve the anti-interference ability and prevent the loss of enzyme molecules [82]. In addition, it is well known that an Ag shell increases electrical conductivity, which is strategically important in electrochemical biosensor applications [78]. Thin Pt (shell) coated on transition metal (core) exhibits significantly improved selectivity, durability, and electrocatalytic activity compared to pure Pt due to structural, electronic, and synergistic interactions between core and Shell [93].

**Fig. 10:**
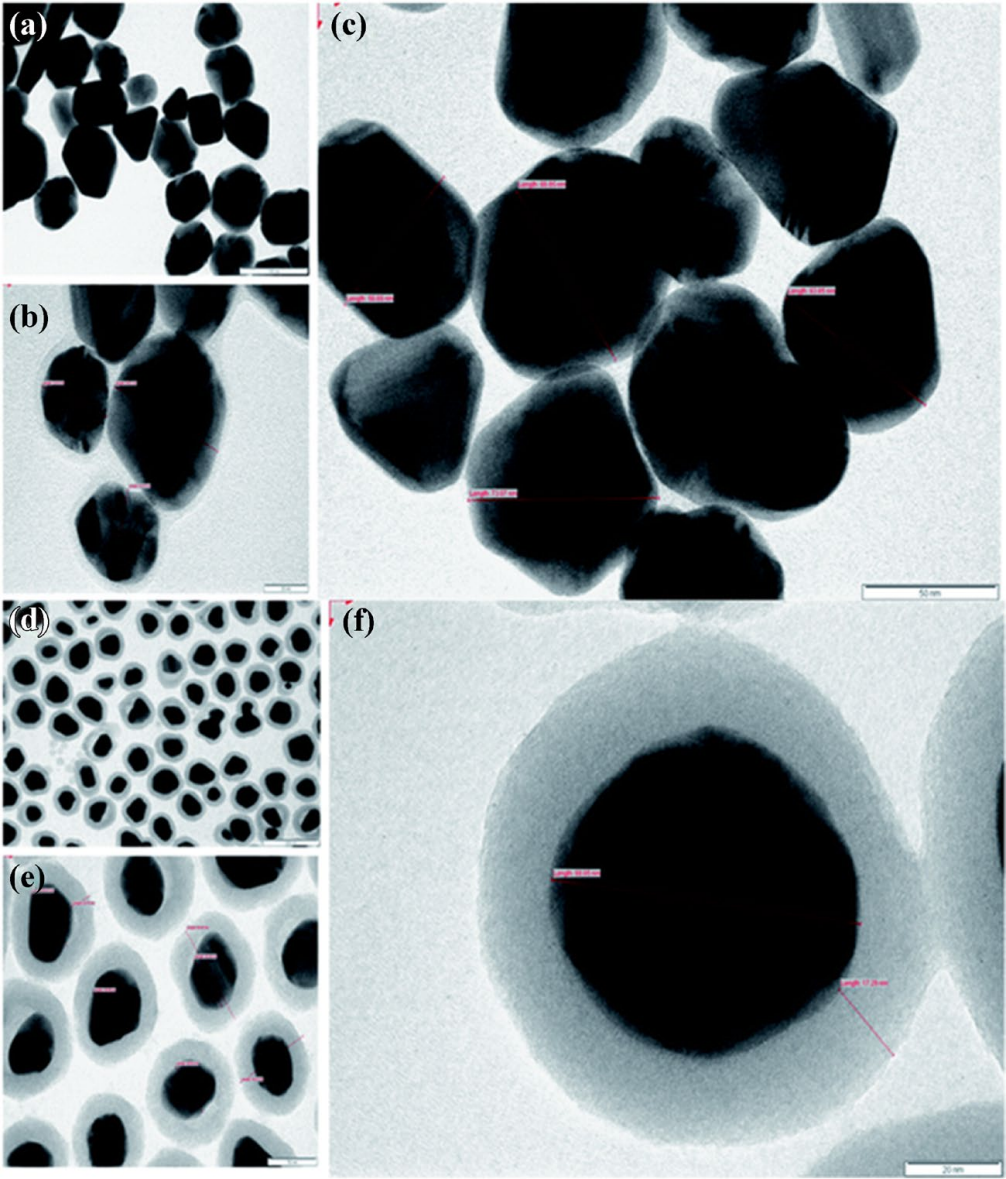
TEM images of the prepared Ag nanoparticles (**a**, **b**, and **c**) and Ag@SiO_2_ nanoparticles (**d**, **e**, and **f**) are shown in the TEM images at different magnifications. Reprinted from Ref. [65] with permission from the Royal Society of Chemistry

Literature surveys show that SiO_2_ shell nanoparticles are used more in enzyme-based biosensor applications. For example, in a study conducted by Singh et al. in 2021, SiO_2_ shell materials were used [65] Furthermore, TEM analysis includes images of Ag@SiO_2_ composite material at different magnifications (Fig. 10). In general, it has been determined that SiO_2_ shells are used in enzymatic glucose determination. The main reason for this is the biocompatibility of SiO_2_ nanoparticles and the increasing stability of magnetic cores [11].

Shell structures can be composed of not only nanoparticle and carbon-like structures but also biological materials. For instance, in a study by He et al., bovine serum albumin was preferred as the shell material [80]. An A glucose sensor was created by immobilizing glucose oxidase (GOx) on poly (methyl methacrylate)-bovine serum albumin (PMMA-BSA) core/shell nanoparticles. The biocompatible BSA protein shell not only immobilized GOx through relatively strong physical interaction but also maintained its bioactivity and native structure, providing thermal stability at room temperature. Thus, with the effect of the shell material, it was observed that the enzyme electrode has a fast response time, low transport barrier, and high affinity for glucose with a wide linear detection range [80].

## Advantages, disadvantages, and limitations of core/shell nanoparticle-based enzyme biosensors

Core/shell nanoparticles serve as protective nanocarriers for enzymes, sheltering them from harsh environmental conditions such as pH differences, temperature changes, and enzymatic degradation [94]. Core/shell nanoparticles are also known as core/shell nanocarriers [95]. Both the structural integrity and the activity of the enzyme are protected by the shell layer, which acts as a physical barrier against the interference of external influences. Core/shell nanoparticle-based biosensors have a longer shelf life than non-nanoparticle-encapsulated biosensors because they minimize enzyme degradation. This improved stability assures constant performance over time, reducing the need for regular recalibration or replacement. This explains that core/shell nanoparticles increase the stability of enzymatic biosensors. Also, core/shell nanoparticles have a large surface area relative to their volume, allowing for the immobilization of a larger concentration of enzymes [96]. This enhanced enzyme loading causes amplified signal production in response to target analytes, increasing the biosensor’s sensitivity. Moreover, core/shell nanoparticles include distinctive surface characteristics, including enhanced surface energy and specialized surface structures, that have the potential to facilitate interactions between enzymes and substrates, hence enhancing catalytic activity [97]. The increase in catalytic efficiency results in enhanced reaction kinetics and strengthened signal amplification, resulting in accelerated response times and lower detection limits. Additionally, core/shell nanoparticle production enables meticulous adjustment of their morphology, dimensions, composition, structure, and surface properties [4]. The capacity to adjust the characteristics of nanoparticles allows researchers to customize them according to specific application needs, such as optimizing the immobilization of enzymes, strengthening the affinity of substrates, or improving the selectivity of sensors. Core/shell nanoparticle-based enzymatic biosensors offer several advantages, including improved stability, sensitivity, catalytic activity, and tunability, making them better candidates than other biosensors for a wide variety of analytical and biomedical applications. However, core/shell nanoparticles have some disadvantages that limit their use. Studies aimed at detecting and overcoming these problems are very important. The preparation of core–shell nanoparticles with precise control over size, content, and surface properties can be difficult and necessitate specialized procedures [98]. This level of intricacy can make scaling and replication difficult. On the other hand, synthesis procedures of core/shell materials include equipment (hydrothermal reactors) that provide controlled temperature and pressure parameters. The use of this equipment causes some disadvantages such as high cost and non-portability. Furthermore, the characterization of these nanoparticles to assure consistency and stability can be time-consuming and expensive. In some cases, the diffusion of substrates and products to and from the active sites of enzymes immobilized on core/shell materials may be hindered by the nanoparticle matrix, which may lead to mass transfer limitations and decreased sensitivity [99]. Although core/shell nanoparticles have a high surface area-to-volume ratio, there may be restrictions to the quantity of enzymes that can be immobilized on them. This limited loading capacity can have an impact on the biosensor’s overall sensitivity and effectiveness, especially in applications that require high enzyme concentrations [100, 101]. Moreover, certain nanoparticle materials may not be biocompatible, which calls for cautious selection and surface changes to reduce toxicity hazards, especially in biological and environmental applications. As mentioned before, Au [82] and Silica [11] materials are generally known to be highly biocompatible in core/shell modification applications. However, to expand the role of core/shell materials in biosensor applications, it is important to synthesize different materials with high biocompatibility. In addition, shell nanoparticles in direct contact with the enzyme may create some disadvantages. The overall performance of the biosensor may be impacted by the presence of the nanoparticle shell, which may interfere with the enzyme’s substrate binding site or change its catalytic characteristics. For this reason, shell nanoparticle selection, design, and observation of the enzyme and its mechanism of action are very important. To overcome these constraints, continued research is needed in the areas of mass transfer characteristics, biocompatibility, interference effects mitigation, synthesis method optimization, enzyme immobilization technique improvement, and cost reduction.

## Concluding remarks

The electrochemical enzyme-based biosensor with real-time, portable, ultra-high sensitivity and selective biosensing is the requirement for practical application in blood sugar, cancer biomarkers, and other organic/inorganic compounds such as pollutants and contaminants. Therefore, the improvement of electrochemical enzyme biosensors based on newly discovered nanomaterials is still growing. It will remain the significant direction for clinical diagnosis and environmental monitoring in the future. The promising core/shell nanomaterials have some advantages, including the shell materials preventing the core nanomaterials from undergoing chemical/physical changes and improving the surface conductivity, stability, and dispersion of core materials. In this review, the benefits of core/shell nanomaterials used for an enzyme-based biosensor in clinical, food, and environmental analysis were discussed. Table 1 summarizes the core–shell modification materials, immobilization technique, electrode types, electrochemical techniques, enzyme and analyte types, application area, linearity range, and LOD for the determination of H_2_O_2_, glucose, and other organic/inorganic compounds. The effects of the properties of the core and shell materials were evaluated together and separately on biosensing processes. The use of core/shell nanomaterials increases the electron transfer between the enzyme and the electrode surface, the development of circumstances of enzyme immobilization and stability, and the catalysis of electrochemical reactions. It is concluded that the GOx enzyme-based electrochemical sensors for determining glucose and iron, Au, and silicon dioxide-containing materials for core/shell modification are commonly studied in biotechnology. The modification of commonly used glassy carbon electrodes with core/shell structure has improved their sensitivity and selectivity. In the future, with the increasing demands for the development of wearable and portable sensors, more attention has been placed on micro/nano-chip devices. For this purpose, the core/shell nanomaterials for biosensors are the best candidates.

**Table 1:** Overview of enzyme-based electrochemical applications and role of core/shell nanoparticles

Electrochemical techniques	Enyzme	Analyte	Core/Shell modification materials	Immobilization techniques	Working electrode	Application	Linearity range	Limit of detection	References
CA	GOx	Glucose	MnO_2_	Cross-linking	SPE	Blood sample	28 µgmL^−1^to 93 µgmL^−1^	7 µgmL^−1^	[45]
A	FAO	HbA_1c_	Fe@Si	Covalent binding	AuE	Blood sample	0 to 2mM	0.1mM	[46]
A	Horseradish peroxidase	H_2_O_2_	organosilica@CHIT	Covalent binding	GCE	Disinfector and sterilized milk samples	7.0 × 10^−7^ to 2.8 × 10^−3^ M	2.5 × 10^−7^ M	[47]
CA	Caspase	miRNA-21	Ag@Au/ GQD	Physical adsorption and covalent binding	PbE	Human plasma	5 pM to 5 mM	5 pM	[35]
EIS	GOx	Glucose	CdTe@CdS/G-AuNP	Covalent binding	AuE	Human saliva samples	1 × 10^−11^ M to 1 × 10^−8^ M	3 × 10^−12^ M	[48]
A	DAO	Histamine	CeO_2_@PANI	Physical adsorption	GCE	Aqueous solution	0.45 to 1.05 mM	48.7 μM	[49]
DPV	GOx	Glucose	Fe_3_O_4_@SiO_2_–Au@mSiO_2_	Physical adsorption	GCE	Aqueous solution	0.01 to 1.11 U mL^−1^	0.004 U mL^−1^	[50]
							11.11 to 476.11 U mL^−1^		
CV	GOx	Glucose	Pd@Pt	Covalent binding	GCE	Blood sample	1 − 6 mM	0.2 μM	[51]
A	HRP	Escherichia coli	Fe_2_O_3_@Au	Physical adsorption	GCE	Bacterial sample	1 × 10^3^ to 5 × 10^5^ cfu/mL	5cfu/mL	[52]
A	HRP	H_2_O_2_	Au@polyaniline	Electrostatic	ITO	Aqueous solution	0.2 to 80μM	0.16μM	[53]
EIS	Laccasa	Dopamine	Fe_3_O_4_@SiO_2_@vmSiO_2_	Covalent binding	GCE	Aqueous solution	1.5–75 μmol L^−1^	0.177 μmol L^−1^	[39]
EIS	AChE	Pesticides	Fe_3_O_4_@MHCS	Physical adsorption	GCE	Fruit samples	0.01 to 100 ppb and 100 to 600 ppb	0.0148 ppb	[54]
							0.01–50 ppb and 50–600 ppb	0.0182 ppb	
CV	HRP	H_2_O_2_	Si@PANI	Physical adsorption	GCE	Rain waters	0.3–8.8 μM	1.8 × 10^−7^ M	[55]
A	GOx	H_2_O_2_	MWCNTs@rGONRs	Physical adsorption	GCE	Pharmacy and meat samples	0.001–1625 μM	0.001 μM	[56]
		NO_2_					0.01–1350 μM	0.01 μM	
CV	HRP	H_2_O_2_	Ag@C	Physical adsorption	ITO	Aqueous solution	5.0 × 10^−7^–1.4 × 10^−4^ M	2.0 × 10^−7^ M	[57]
A	HRP	H_2_O_2_	Fe_3_O_4_@poly(dopamine)	Physical adsorption	GCE	Aqueous solution	6.0 × 10^−7^to 8.0 × 10^−4^ M	182 nM	[58]
A	MA	IgG	Ag@Au	Cross-linking	PMEs	Human serum	2.3 to 960 ng mL^−1^	10 ng mL^−1^	[59]
EIS	DAO	Salmonella	CeO_2_-NR@Ppy	Covalent binding	Redox probe	Aqueous solution	0.01–0.4 nM	0.28 nM	[40]
A	GOx	Glucose	Fe_3_O_4_@SiO_2_	Covalent binding	CPE	Drink samples	0.25– 2.0 mM	-	[60]
CV	GOx	Glucose	PDDA–Fe_3_O_4_@Au	Magnetic force	MGCE	Human serum	0.02 to 1.875 mM	6.5 μM	[61]
A	HRP	H_2_O_2_	Fe_3_O_4_@NMCMs	Physical adsorption	GCE	Milk samples	50 μMto 33mM	5.9 μM	[62]
DPV	Laccasa	BPA	CuF@HA	Physical adsorption	GCE	Milk samples	0.01–7.50 μM	5.40 nM	[63]
Potentiometric	GOx	Glucose	ZnO@CHIT-g-PVA	Electrostatic	ITO	Human blood serum and urine	2 μM to 1.2 mM	0.2 μM	[64]
Potentiometric EIS	HRP	CEA	Si@Ag	Electrostatic	ITO	Aqueous solution	0.5 to 10 ng mL^−1^	0.01 ng mL^−1^	[65]
EIS	HQ hydroq	H_2_O_2_	Fe_3_O_4_@CHIT	Cross-linking	GCE	Real samples	5.0 × 10^–5^ to 1.8 × 10^–3^ M	4.0 × 10^–6^ M	[66]
							1.8 × 10^–3^ to 6.8 × 10^–3^ M		
PEC	BChE	Pesticides	MnO_2_ nanoflower@CdS	-	ITO	Red wine and milk samples	-	0.68 pg mL^−1^	[67]
CV	GOx	Glucose	ZnO@ZnS	Cross-linking	Nanotube arrays	Aqueous solution	2.39 × 10^−5^ to 2.66 × 10^−4^ mM	24 μM	[68]
EIS	GOx	Glucose	Fe_3_O_4_@silica@Au	Physical adsorption	GCE	Aqueous solution	-	3.97mM	[69]
A	GOx	Glucose	Au@Ag–Pt@ICPs	Physical adsorption	GCE	Blood sample	0.5 μM to 3.33 mM	60 nM	[70]
A	Tyr	Phenol	Fe_3_O_4_@mSiO_2_	Cross-linking	GCE	Real samples	1.0 × 10^−9^ to 1.0 × 10^−5^ M	1nM	[9]
A	HRP	H_2_O_2_	TiO_2_	Redoks immobilization	GCE	Aqueous solution	0.4–140 μM	0.05 μM	[36]
PEC	GOx	Glucose	TiO_2_@PDA -NR	Physical adsorption	FE	Aqueous solution	0.2– 1 mM	0.0285 mM	[71]
A	GOx	Glucose	AgCl@PANI	Physical adsorption	GCE	Ascorbic acid,uric acid, cysteine	4–34 pM	4 pM	[72]
CA	GOx	Glucose	3D AgNCs@PB	Cross-linking	SPE	Rabbit serum samples	0.01mM to 1.3 mM	0.005 mM	[73]
A	GOx	Glucose	Au@Ag NRs	Physical adsorption	GCE	Blood sugar in a real serum	0.02 to 7.02 mM	0.67 μM	[74]
A	GOx	Glucose	Fe_3_O_4_–enzyme@Ppy	Magnetic force	MGCE	Human serum	0.5 μM to 34 mM	0.3 μM	[41]
CA	Laccase	Hydroquinone	Fe_3_O_4_@SiO_2_	Covalent binding	CPE	Vanillin, 3,5-dinitrosalicylic acid, guaiacol	1 × 10^−7^ to 1.375 × 10^−4^ M	1.5 × 10^−8^ M	[75]
DPV	HRP	H_2_O_2_	CdTe@CdS QD	Physical adsorption	AuE	Real samples	1 × 10^−10^ M to 1.2 × 10^−8^ M	3.2 × 10^−11^ M	[76]
CV	GOx	Glucose	Ag@C	Physical adsorption	GCE	Aqueous solution	0.05–2.5 mM	0.02 mM	[77]
CV	HRP	Cholesterol	Fe_3_O_4_@C@Ag	Covalent binding	ITO	Blood serum	0.5 to 22.5 mM	0.5 mM	[78]
	ChOx								
A	Con A	Brett	Fe_3_O_4_@SiO_2_	Covalent binding	SPE	Red wine	0 – 10^–6^ CFUmL^−1^	5CFU mL^−1^	[37]
A	AChE	Pesticides	Pd@Au	Physical adsorption	GCE	Tap water samples	3.6 pM–100 nM	3.6 pM	[79]
CA	GOx	Glucose	PMMA@BSA	Cross-linking	PDE	Human serum	0.2 to 9.1 mM	-	[80]
DPV	GOx	TB	Au@Pd	Covalent binding	GCE	Human serum	0.1 pM to 30 nM	0.037 pM	[81]
CA	GOx	Glucose	Au–Fe_3_O_4_@SiO_2_	Cross-linking	ITO	Human serum	0.05–1.0 mM	0.01 mM	[82]
							1.0–8.0 mM		
EIS	AChE	Pesticides	AuNRs@MS	Physical adsorption	GCE	Vegetable juice samples	0.018 μM to 13.6 μM	5.3 nM	[38]
A	AChE	Parathion methyl	PtPd@NCS	Covalent binding	GCE	Potato and corn grain sample	1 × 10^–14^ to 1 × 10^–11^ M and 1 × 10^−10^ to 1 × 10^−5^ M	8.6 × 10^–15^ M	[42]
		Chlopyrifos					1 × 10^–13^ to 1 × 10^−6^ M	7.1 × 10^–14^ M	
		Malathion					1 × 10^–14^ to 1 × 10^−10^ M and 1 × 10^−9^ to 1 × 10^−5^ M	7.9 × 10^–15^ M	
A	GOx	Glucose	ZnO nanotubes@MnO_2_	Covalent binding	3DGNE	Aqueous solution	1 µM to 0.07 mM	10 nM	[83]
DPV	AChE	Malathion	COF@MWCNTs	Physical adsorption	GCE	Tap water	1 nM to 10 μM	0.5 nM	[84]
DPV	Tyr	DA	ZnO@Au	Physical adsorption	SPE	Synthetic urine	0.1 to 500 μmol L^−1^	86 nmol L^−1^	[85]
DPV		T4 PNK	Fe_3_O_4_@TiO_2_	Physical adsorption	AuE	-	0.0001 to 10 U mL^−1^	0.00003 mL^−1^	[86]

## Data Availability

Data will be available on request.

## Abbreviations

A: Amperometric
AChE: Acetylcholinesterase
AuE: Gold electrode
AuNRs: Au nanorods
BChE: Butyrylcholinesterase
BET: Brunauer–Emmett–Teller
BPA: Bisphenol A
Brett: Brettanomyces bruxellensis
BSA: Bovine serum albumin
C: Carbon
^13^C NMR: Carbon-13 nuclear magnetic resonance
CA: Chronoamperometry
CEA: Carcinoembryonic antigen
CHIT: Chitosan
ChOx: Cholesterol oxidase
CPE: Carbon paste electrode
COF@MWCNTs: Covalent organic framework@multi-walled carbon nanotubes
Con A: Concanavalin A
CV: Cyclic voltammetry
CVD: Chemical vapor deposition
DA: Dopamine
DAO: Diamine oxidase
ECL: Electrochemiluminescence
EDX: Energy dispersive X-ray
EIS: Electrochemical impedance spectroscopy
FADH: Flavin adenine dinucleotide
FAO: Fructosyl amino-acid oxidase
FE: Fabricated electrode
FT-IR: Fourier-transform infrared spectroscopy
g-PVA: Graft-poly (vinyl alcohol)
G-AuNP: Graphene-gold nanocomposite
GCE: Glass carbon electrode
GQD: Graphene quantum dots
GOx: Glucose oxidase enzyme
HA: Hyaluronic Acid
HbA1c: Glycosylated hemoglobin
HMSNs: Hollow porous silica nanoparticles
HRP: Horseradish peroxidase
ICP: Infinite coordination polymer
ITO: Indium–tin–oxide
LOD: Limit of detection
LSV: Linear sweep voltammetry
MA: Monoamine oxidase
MGCE: Magnetic glassy carbon electrode
MHCS: Mesoporous hollow carbon spheres
MS: Mesoporous silica
MWCNTs: Multiwalled carbon nanotubes
NADH: Nicotinamide adenine dinucleotide
NCS: N-doped carbon shell
NMCMs: Nitrogen-doped mesoporous carbon microcapsules
NR: Nanorod
PANI: Polyaniline
PB: Prussian blue
PDA: Poly-dopamine
PEC: Photoelectrochemical
PbE: Paper-based electrode
PMEs: Platinum microelectrodes
PMMA: Poly (methyl methacrylate)
PDE: Platinum disk electrode
PPy: Polypyrrole
QD: Quantum dots
rGONRs: Reduced graphene oxide nanoribbons
SEM: Scanning electron microscopy
SPE: Screen-printing electrode
TB: Thrombin
TEM: Transmission electron microscopy
T4 PNK: T4 polynucleotide kinase
Tyr: Tyrosinase
XFELs: X-ray free electron lasers
XRD: X-ray diffraction analysis
ZIF-8: Zeolitic imidazolate framework
3DGNE: 3D graphene network electrode
